# Phylogeny, Antifungal Susceptibility, and Point Mutations of *SQLE* Gene in Major Pathogenic Dermatophytes Isolated From Clinical Dermatophytosis

**DOI:** 10.3389/fcimb.2022.851769

**Published:** 2022-03-18

**Authors:** Nasrin Pashootan, Masoomeh Shams-Ghahfarokhi, Arash Chaichi Nusrati, Zahra Salehi, Mehdi Asmar, Mehdi Razzaghi-Abyaneh

**Affiliations:** ^1^Department of Microbiology, Faculty of Basic Sciences, Lahijan Branch, Islamic Azad University, Lahijan, Iran; ^2^Department of Mycology, Faculty of Medical Sciences, Tarbiat Modares University, Tehran, Iran; ^3^Department of Mycology, Pasteur Institute of Iran, Tehran, Iran

**Keywords:** dermatophytes, antifungal susceptibility testing, point mutation, terbinafine resistance, Phe397Leu substitution, *T. indotineae*, dermatophytosis, squalene epoxidase

## Abstract

Drug resistance is one of the major challenges to skin fungal infections, especially in tropical and subtropical infections caused by dermatophytes. This study aimed to determine the antifungal susceptibility of clinically dermatophytes and evaluate point mutations in terbinafine-resistant isolates. A total number of 123 clinical dermatophyte isolates in eight species were evaluated in terms of sensitivity to seven major antifungals. Furthermore, the point mutation in squalene epoxidase (*SQLE*) gene responsible for terbinafine resistance was studied. The dermatophytes species were identified by morphological characteristics and confirmed by the ITS sequencing. Also, the phylogenetic tree was drawn using the RAxML analyses for 123 dermatophytes isolates. A new XXIX genotype was also found in 4 *Trichophyton mentagrophytes* isolates. Based on the results obtained, terbinafine was the most effective antifungal drug followed by itraconazole and voriconazole. *Trichophyton rubrum* and *Trichophyton tonsurans* were the most susceptible species (MIC_50_ = 0.01, 0.09 μg/ml), and *T. mentagrophytes* was the most resistant species (MIC_50_ = 0.125 μg/ml) to terbinafine. Of the 123 dermatophytes isolates, six isolates showed reduced susceptibility to terbinafine, and only *Trichophyton indotineae* had a mutation in *SQLE* gene as a Phe397Leu substitution. Overall, the antifungal susceptibility test is necessary for managing dermatophytosis. These results help physicians to control the course of the disease and provide further insights to select effective drugs for patients with dermatophytosis, especially in tropical and subtropical regions of the world, where dermatophytosis is still a public health problem.

## 1 Introduction

Dermatophytes are a group of keratinophilic fungi with considerable morphological and genetic similarities (Dabas et al., 2017; Salehi et al., 2021). They can attack keratinized tissue of humans and animals and create dermatophytosis (Zareshahrabadi et al., 2020). According to the new classification, dermatophytes are classified into *Trichophyton*, *Epidermophyton*, *Nannizzia*, *Paraphyton*, *Lophophyton*, *Microsporum*, and *Arthroderma* (de Hoog et al., 2017).

According to the WHO’s evaluation, dermatophytosis affects 20%–25% of the world population (Ebert et al., 2020). In addition, studies have shown that dermatophyte species responses to antifungal drugs are not the same (Bhatia and Sharma, 2015). The confirmed therapeutic strategies for dermatophytosis infection include griseofulvin (GRI) drugs and systemic or topical triazole and allylamine drugs, which mostly include itraconazole (ITZ) and terbinafine (TRB). Currently, TRB is the first choice for the treatment of dermatophytosis given its stable clinical effect and fewer recurrence (Haugh et al., 2000; Niimi et al., 2010). On the other hand, therapeutic failure has become an alarming trend, and health systems around the world pay heavy costs to treat the disease every year (Siopi et al., 2021). In addition, there are an increased number of studies about resistance to antifungal drugs and TRB in particular in dermatophyte species (Haugh et al., 2000; Mukherjee et al., 2003; Osborne et al., 2005; Niimi et al., 2010; Singh et al., 2018; Nenoff et al., 2020; Siopi et al., 2021).

The TRB is a synthetic allylamine derivative that, by the activity of squalene epoxidase (*SQLE*), causes the accumulation of squalene and decreases ergosterol of the cell membrane, which leads to cellular death (Ghannoum et al., 2004; Osborne et al., 2005; Afshari et al., 2016).

The genetic basis of TRB resistance in fungi including *Aspergillus fumigatus*, *Aspergillus nidulans*, and *Trichophyton rubrum* has shown that the resistance may arise from the overexpression of *SQLE* gene, or it could result from mutations in other genes that indirectly affect antimycotic susceptibility (Rocha et al., 2002; Liu et al., 2004; Santos et al., 2018). It has been shown that resistance to TRB in dermatophyte species is related to the mutation of *salicylate 1-monooxygenase* (*sa1A*) and *SQLE* (Mukherjee et al., 2003; Yamada et al., 2017). Studies have shown that the resistance is more related to the substitutions at one of the amino acid positions of Leu393, Phe397, Phe415, and His440 in *SQLE* protein (Mukherjee et al., 2003; Osborne et al., 2005; Osborne et al., 2006). Furthermore, a recent study by Nenoff et al. (2020) identified two substitutions of amino acids at Ser395Pro and Ser443Pro positions of *SQLE* in *Trichophyton* strains resistant to TRB. It has been shown that replacement in the F397L position of *SQLE* is the most common type of substitution. There are reports of mutation in different positions of *SQLE* in *T. tonsurans*, *T. interdigitale*, *T. mentagrophytes,*, and *T. rubrum* isolates resistant to TRB (Rocha et al., 2002; Singh et al., 2018). In addition, Taghipour et al. showed that there was a relationship between *SQLE* mutation in the species resistant to TRB and ITS genotype. So the resistant strains of *T. mentagrophytes* are categorized only in VIII genotype, and resistant species of *T. interdigitale* are categorized in II genotype. On the other hand, according to the new classification of *T. mentagrophytes*, subtype VIII is considered as a separate species named *Trichophyton indotineae* (Kano et al., 2020).

However, the emergence of TRB-resistant dermatophyte strains in the south of Asia and an increase in resistance of the strains to antifungal drugs are survival mechanisms of dermatophyte fungi, which lead to failure of treatment and recurrence of disease (Ebert et al., 2020). Therefore, in this study, to accurately identify dermatophyte species, ITS region sequence was utilized, and ITS genotypes of *T. mentagrophytes* and *T. interdigitale* were determined. In addition, antifungal activity assessment of TRB drugs, ITZ, ketoconazole (KTZ), fluconazole (FLZ), posaconazole (PCZ), voriconazole (VCZ), and amphotericin B (AMB) was done through Clinical and Laboratory Standards Institute (CLSI) broth microdilution M38-A2 method against 123 clinical isolates and five standard isolates of dermatophyte. In addition, the point mutation in *SQLE* gene was investigated in TRB-resistant strains.

## 2 Material and Methods

### 2.1 Clinical Fungal Isolation

This experimental study included 123 dermatophyte isolates obtained from the patients visiting the Mycology Department, Pasteur Institute of Iran, between 2018 and 2019. The isolates were identified using microscope and culture, and for final confirmation, ITS sequence was used. In addition, five standard strains were provided from Persian Type Culture Collection (PTCC), Iranian Research Organization for Science and Technology (Karaj-Iran), including *T. mentagrophytes* PTCC 5054, *Microsporum canis* PTCC 5069, *Nannizzia gypsea* PTCC 130396, *Trichophyton verrucosum* PTCC 10694, and *T. rubrum* PTCC 5808, which were used as quality control. The species under study were *T. mentagrophytes/T. interdigitale* complex (include *T. indotineae*), *T. rubrum*, *T. tonsurans*, *Epidermophyton floccosum*, *T. verrucosum*, *N. gypsea*, *Nannizzia fulva*, and *M. canis* (**Table 1**).

**Table 1:** Clinical features of the 123 clinical dermatophyte strains based on the infection body area.

Clinical manifestation	Etiologic agents, no. (%)										
	*Trichophyton mentagrophytes*	*Trichophyton interdigitale*	*Trichophyton indotineae*	*Trichophyton rubrum*	*Trichophyton tonsurans*	*Trichophyton verrucosum*	*Epidermophyton floccosum*	*Microsporum canis*	*Nannizzia gypsea*	*Nannizzia fulva*	Total no. (%)
Tinea cruris	2 (1.62)	11 (8.84)	5 (4.06)	5 (4.06)	3 (2.43)	–	14 (11.38)	1 (0.81)	–	–	41 (33.33)
Tinea pedis	2 (1.62)	9 (7.31)	3 (2.43)	7 (5.69)	–	–	3 (2.43)	–	–	–	24 (19.51)
Tinea corporis	1 (0.81)	1 (0.81)	2 (1.62)	–	2 (1.62)	–	1 (0.81)	5 (4.06)	–	–	12 (9.75)
Tinea capitis	–	2 (1.62)	–	–	8 (6.50)	–	–	4 (3.25)	2 (1.62)	–	16 (13.00)
Tinea unguium	–	–	–	–	–	–	–	–	4 (3.25)	2 (1.62)	6 (4.87)
Tinea faciei	–	–	–	–	3 (2.43)	5 (4.06)	–	–	–	–	8 (6.50)
Tinea manuum	1 (0.81)	5 (4.06)	–	3 (2.43)	–	2 (1.62)	1 (0.81)	4 (3.25)	–	–	16 (13.00)
Total	6	28	10	15	16	7	19	14	6	2	123

### 2.2 Molecular Identification by ITS Region

All fungal strains were cultured on Mycobiotic agar (Merck, Darmstadt, Germany) and incubated at 27°C for 7 days (Osborne et al., 2005). In summary, fungal cell fragmentation was performed by liquid nitrogen and added to the extracted cells of DNA extraction buffer containing 200 M of Tris-HCl, pH 8, 25 mM of EDTA, SDS 0.5% W/V, and NaCl 250 mM. The DNA extraction method was based on phenol/chloroform/isoamyl alcohol (25:24:1) and proteinase K. After extraction, the resulting DNA was re-dissolved in 50 μl of Tris-EDTA (TE) buffer and stored at −20°C (Salehi et al., 2018a). The ITS region was PCR amplified using primers ITS1 (5′-TCCGTAGGTGAACCTGCGG-3′) and ITS4 (5′-TCCTCCGCTTATTGATATGC-3′) (White et al., 1990). The final volume of PCRs was 25 μl, containing 12.5 μl of premix (Ampliqon, Odense, Denmark), 1 μl of DNA template, 0.5 μM of forward and reverse primers, and distilled water. The PCR cycling conditions were as follows: 5 min initial pre-incubation at 95°C, followed by 35 cycles consisting of denaturation at 94°C for 30 s, annealing at 58°C for 30 s, and extension at 72°C for 45 s, with a final extension at 72°C for 5 min (Salehi et al., 2020). Five microliters of the PCR products was electrophoresed on the 1% agarose gel in Tris/Borate/EDTA (TBE) buffer (Yurkov et al., 2012). The sequences of isolates were edited manually and subjected to ClustalW pairwise alignment using the MEGA10 software. The sequences deposited in GenBank are shown in **Table 2**. ITS genotyping determined *T. interdigitale/T. mentagrophytes* species complex according to the studies by Heidemann et al. (2010) and Taghipour et al. (2019). In addition to examining the relationship between the genotype and resistance to TRB, the ITS genotype of complex isolates *T. mentagrophytes*/*T. interdigitale*/*T. indotineae* was determined.

**Table 2:** Age and sex distribution of dermatophyte species isolated from clinical specimens.

Dermatophytes species	Sex (no.)		Age (no.)								Accession no.
	F	M	0–10	11–20	21–30	31–40	41–50	51–60	61–70	71–80	
*Trichophyton mentagrophytes* (n = 6)	4	2	1	–	2	1	1	1	–	–	MZ983790- MZ983485- MZ994488- MZ994650- MZ994652- MZ994491
*Trichophyton interdigitale* (n = 28)	12	16	1	–	3	11	5	6	2	–	OK 35221- OK 35220- OK 110565- OK 110585- OK 110591- OK 110574- OK 110568- OK 110566- OK 110586- OK 110567- OK 110580- OK 110572- OK 110578- OK 110576- OK 110577- OK 35231- OK 110583- OK 110570- OK 110569- OK 110589- OK 110571- OK 35266-o OK 110575- OK 110581- OK 110573- OK 110579- OK 110587-OK110582
*Trichophyton indotineae* (n = 10)	4	6	–	1	1	5	2	1	–	–	OM366332 to OM366341
*Trichophyton rubrum* (n = 15)	3	12	–	–	2	2	7	1	2	1	MT188699- MZ427314- MZ434885- MT188700- MT150739- MZ434887- MZ434886- MZ427316- MT191357- MT152325
*Trichophyton tonsurans* (n = 16)	3	13	4	6	3	1	2	–	–	–	MT041242- MT041041- MT041256- MT066197- MT051844
*Trichophyton verrucosum* (n = 7)	–	7	–	–	2	2	2	1	–	–	MT318679- MT318720
*Epidermophyton floccosum* (n = 19)	5	14	1	–	4	6	3	2	3	–	MT040969- MT040750- MT150728- MT040755- MZ363671- MT040763- MZ363673- MZ363722- MZ363721- MT040762- MZ363674
*Microsporum canis* (n = 14)	9	5	4	2	3	3	–	2	–	–	MT129526- MT067649- MT183698- Mz363857- MT136105- MT129500
*Nannizzia gypsea* (n = 6)	3	3	2	2	1	–	–	1	–	–	MT318651- MZ434959- MZ435310- MT394865
*Nannizzia fulva* (n = 2)	2	–	1	1	–	–	–	–	–	–	–

Then, the sequences were analyzed by RAxML version 8.2 (Stamatakis, 2014) running on CIPRES Science Gateway (Miller et al., 2010). Optimization in RAxML was carried out using the GTRCAT option. Bootstrap values for maximum likelihood were 1,000 replicates with one search replicate per bootstrap replicate and *Fusarium solani* as the outgroup.

### 2.3 Antifungal Susceptibility Testing

#### 2.3.1 Chemical Antifungal Drugs

The drug susceptibility test was performed through minimum inhibitory concentration (MIC) microdilution broth. The drugs related to the MIC test were prepared according to the M38-A2 protocol for filamentous fungi (Wayne, 2008).

#### 2.3.2 Drug Susceptibility Testing Using Microdilution Broth

The broth microdilution was used following M38-A2 CLSI protocol to examine and assess MIC in all strains (Wayne, 2008). According to the CLSI standard, drug stocks were prepared in dimethyl sulfoxide (DMSO). Different concentrations (100 µl) were poured into 96-well round-bottom microplates from the lowest concentration to the highest concentration. According to the CLSI standard, the range of antifungals was as follows: 0.001–32 μg/ml for TRB; 0.01–16 μg/ml for ITZ, KTZ, VCZ, PCZ, and AMB; and 0.06–64 μg/ml for FLZ. Then the prepared suspensions (100 µl) of each strain containing 1-3 × 10^3^ ml/CFU spore were added to the wells. The plates were incubated at 35°C and visually assessed for fungal growth after 96 h. The MIC range, geometric mean, MIC_50_, and MIC_90_ were calculated for all the isolates tested.

### 2.4 PCR Assay Targeting the SQLE Region

To investigate mutations in *SQLE* gene, the strains with less susceptibility to TRB were evaluated with the primers Drsq1 (5′-TTGCCAACGGGGTGTAAAG-3′) and Drsq2 (5′-GGGGCCATCTATAATTCAGACTC-3′) (Osborne et al., 2006). The primer for replacing amino acids in Leu393Phe, Leu393Ser, Phe397Leu, and Gln408Leu in *SQLE* was used. The length of the fragments for *Trichophyton* was 500 bp, while it was 520 bp for *Epidermophyton* and *Nannizzia*. According to CLSI, *T. rubrum* strains with MIC > 0.5 µg/ml and other strains with MIC > 0.25 µg/ml were selected as the strains with less sensitivity to TRB.

The PCRs were prepared with the final volume of 50 μl containing 25 μl of premix (Ampliqon, Denmark), 3 μl of DNA template, 0.6 μM of forward and reverse primers, and distilled water. The PCR cycling conditions were as follows: 5 min initial pre-incubation at 95°C, followed by 35 cycles consisting of denaturation at 94°C for 30 s, annealing at 58°C for 30 s, and extension at 72°C for 45 s, with a final extension at 72°C for 5 min (Salehi et al., 2018b). The PCR products of the *SQLE* region were sequenced by the ABI PRISM BigDye Terminator Cycle Sequencing Ready Reaction Kit. The forward and reverse sequences of isolates showing reduced susceptibility to TRB were subjected to ClustalW pairwise alignment using the MEGA10 software and edited manually to improve the alignment accuracy.

### 2.5 Statistical Analysis

The quantitative data from MIC, the geometric mean, MIC_50_, and MIC_90_ were calculated using the SPSS statistical package.

## 3 Results

### 3.1 Characteristics of the Studied Dermatophyte Isolates

Clinical data of 123 patients including 82 men (66.66%) and 41 women (33.33%) are listed in **Table 1**. In addition, the age range of the patients was 2–80 years with a mean age of 41. The most common clinical manifestations were tinea cruris (34.95%) followed by tinea pedis (17.88%), tinea manuum (19.51%), and tinea capitis (16.26%). The dominant species in tinea cruris, tinea pedis, and tinea capitis were *T. tonsurans*, *T. rubrum*, and *E. floccosum*, respectively. In addition, the isolated species from tinea faciei only included *T. tonsurans* and *T. verrucosum*. It is significant that *N. fulva* was only isolated from tinea unguium and that *T. verrucosum* was only isolated from the male cases.

### 3.2 Molecular Identification by ITS Region

**Figure 1:**
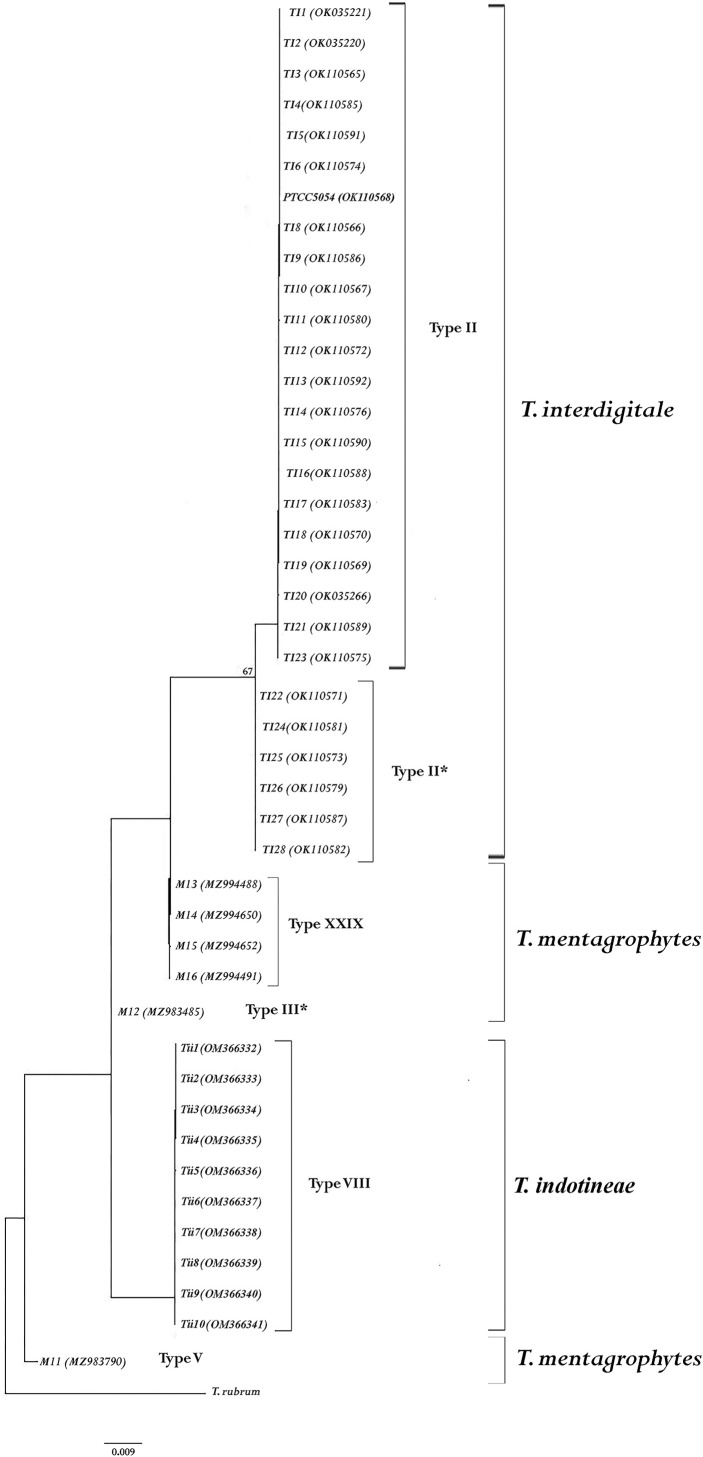
The genotyping tree was constructed based on ITS region sequences by RAxML analysis from *Trichophyton mentagrophytes/Trichophyton interdigitale* complex, and the accession numbers are given in parentheses*. Trichophyton rubrum* was used as an outgroup to root the dendrogram. The bootstrap value greater than 65% is shown above the branches.

All the dermatophyte species were identified by determining the sequence of ITS-rDNA regions. The results of the molecular method indicated species *T. mentagrophytes* (6), *T. interdigitale* (28), *T. indotineae* (10), *E. floccosum* (19), *T. tonsurans* (16), *T. rubrum* (15), *M. canis* (14), *T. verrucosum* (7), *N. gypsea* (6), and *N. fulva* (2). After manually editing and blast analysis, the sequences were deposited in GenBank (**Table 2**). In addition, ITS genotype examination results showed that out of 6 *T. mentagrophytes* isolates, there was one isolate in each of the V and III* genotypes. *T. indotineae* was located between genotypes V and III* of *T. mentagrophytes*. Also, a new genotype was found in four isolates of *T. mentagrophytes* (XXIX). *T. interdigitale* isolates were only seen in two genotypes II (n = 22) and II* (n = 6) (**Figure 1**).

The phylogenetic tree was drawn through RAxML analysis for all species (**Figure S1**). The tree illustrates the phylogenetic relationships between all isolates under study. Based on phylogenetic analysis, all isolates were placed in six clades (*T. mentagrophytes*, *T. interdigitale*, and *T. indotineae* were in separate clusters). In addition, *N. gypsea* and *N. fulva* were observed in one clade with high support. In addition, the *M. canis* clade was in the base position compared to other species (**Figure S1**). Supports of bootstrap higher than 85% were represented in RAxML analysis. In addition, the phylogenetic tree did not demonstrate a relationship between the isolates that showed a decrease in sensitivity to TRB.

### 3.3 Antifungal Susceptibility Testing

MIC range, geometric mean MIC, MIC_50_, and MIC_90_ were computed for all dermatophyte strains (**Table 2**). In general, among all strains, the highest antifungal effects were observed with TRB followed by ITZ, VCZ, KTZ, FLZ, AMB, and PCZ. The most sensitive strains to TRB were *T. rubrum*, *T. tonsurans*, *T. interdigitale*, *M. canis*, *T. verrucosum*, *N. gypsea*, *N. fulva*, *T. mentagrophytes*, *E. floccosum*, and *T. indotineae* in a descending order. In addition, the results showed that *M. canis* (MIC_50_ = 0.12 μg/ml) had the highest MIC and *T. indotineae* (MIC_50_ = 1 μg/ml) had the lowest MIC against ITZ. In addition, KTZ had a low suppressive effect only against *E. floccosum* (G mean = 1.07 μg/ml). Also, among *T. mentagrophytes*, *T. interdigitale*, and *T. indotineae* species, the lowest and highest sensitivity to seven antifungal drugs tested was observed in *T. indotineae* and *T. interdigitale*, respectively. Furthermore, TRB had the highest antifungal effects against *T. indotineae*.

Moreover, VCZ had a high suppressive effect on *N. gypsea* species. The FLZ had a lower suppressive effect on *T. rubrum* and *T. mentagrophytes* species compared to other species. In general, the lowest suppressive effect on all dermatophyte species was observed with PCZ (**Table 3**).

**Table 3:** Antifungal susceptibility profile of 123 dermatophyte strains to seven antifungal agents by broth microdilution method.

Dermatophyte species	Antifungal drugs	MIC range (µg/ml)	MIC_50_/MIC_90_ (µg/ml)	G mean (µg/ml)	MIC (µg/ml)															
					64	32	16	8	4	2	1	0.5	0.25	0.125	0.06	0.03	0.015	0.007	0.003	0.001
*Trichophyton mentagrophytes* (n = 6)	TRB	0.015–16	0.09/–	0.13			1							2	1	1	1			
	ITZ	0.125–8	0.75/–	0.70				1			2	1	1	1						
	KTZ	0.06–8	1.25/–	1				1	1	1		1	1	1						
	FLZ	0.125–16	1.25/–	1.2			1		1	1		1	1	1						
	VCZ	0.125–8	0.3/–	0.5				1			1	1	1	2						
	PCZ	0.125–16	1.25/–	1.09			1	1		1		1	1	1						
	AMB	0.125–8	0.75/–	0.79				1			2	1	1	1						
*Trichophyton interdigitale* (n = 28)	TRB	0.003–0.025	0.06/0.125	0.04										7	8	6	4	2	1	
	ITZ	0.03–16	0.25/8.8	0.38			2	1	2	2	1	4	4	9	2	1				
	KTZ	0.03–16	0.5/8.8	0.37			2	1	2	1	3	6	2	7	2	2				
	FLZ	0.125–64	0.25/35.2	0.78	2	1	2	2	1	1	1	3	4	10						
	VCZ	0.125–16	0.37/16	0.70			4	3		1	2	4	6	8						
	PCZ	1–16	1.5/16	1.55			6	3	3	2	4	3	2	4	1					
	AMB	0.125–8	0.25/8	0.56				5	2		3	3	6	9						
*Trichophyton indotineae* (n = 10)	TRB	0.015–32	0.125/30.4	0.24		1	1							5	1	1	1			
	ITZ	0.06–16	1/15.2	0.9			1		1	1	2		1	3	1					
	KTZ	0.125–16	0.5/16	0.79			2		2			2	2	2						
	FLZ	0.125–16	0.75/14.8	0.93			1		3		1	1	2	2						
	VCZ	0.125–8	0.37/7.4	0.53				1		1	2	1	3	2						
	PCZ	0.125–16	0.75/16	1			2		1	1	1	1	2	2						
	AMB	0.125–16	0.5/15.2	0.81			1	1		1	1	2	3	1						
*Trichophyton rubrum* (n = 15)	TRB	0.003–0.006	0.015/0.06	0.01											3	2	3	2	5	
	ITZ	0.06–16	0.25/14	0.30			1			1	1	2	3	2	5					
	KTZ	0.125–16	0.25/16	0.75			2	2	1			2	4	4						
	FLZ	0.125–16	0.5/16	1.04			3	1	1	–	2	2	4	2						
	VCZ	0.06–8	0.25/5.6	0.39				1	1	1	1	3	2	5	1					
	PCZ	0.25–16	1/16	1.58			3	1	1	1	3	4	2							
	AMB	0.125–8	0.5/8	0.79				3	1	1	2	2	3	3						
*Trichophyton tonsurans* (n = 16)	TRB	0.003–0.125	0.09/0.125	0.07										8	5	3				
	ITZ	0.06–8	0.37/8	0.51				2	2	1	1	2	2	4	2					
	KTZ	0.125–16	0.5/10.4	0.64			1	2			2	4	5	2						
	FLZ	0.125–32	0.5/20.8	0.67		1	1			1	2	5	4	2						
	VCZ	0.06–16	0.25/16	0.49			3	1				2	3	5	2					
	PCZ	0.125–16	0.75/16	1.24			4	2			2	2	3	3						
	AMB	0.06–16	0.25/16	0.56			3	2				2	3	2	3	1				
*Trichophyton verrucosum* (n = 7)	TRB	0.06–4	0.125/–	0.16					1					4	2					
	ITZ	0.06–1	0.25/–	0.30							2	1	2	1	1					
	KTZ	0.125–1	0.5/–	0.37							2	2	1	2						
	FLZ	0.125–16	0.5/–	0.90			1	1		1		1	1	2						
	VCZ	0.03–16	0.5/–	0.40			1	1			2			1	1	1				
	PCZ	0.125–16	0.5/–	1.21			2		1			1	2	1						
	AMB	0.06–2	0.25/–	0.30						1	1	1	1	2	1					
*Epidermophyton floccosum* (n = 19)	TRB	0.06–16	0.125/0.25	0.17			1						6	9	3					
	ITZ	0.06–16	0.5/16	0.80			3	2		1	2	2	5	3	1					
	KTZ	0.125–16	0.5/16	1.07			4	2	1	1	1	3	3	5						
	FLZ	0.125–32	0.37/32	0.77		2	2			1	1	3	7	3						
	VCZ	0.03–8	0.5/8	0.80				5	1	2		3	3	4	1					
	PCZ	0.125–16	0.5/16	1			3	2	1	1	2	3	4	3						
	AMB	0.06–16	0.25/16	0.57			3	2				4	3	5	2					
*Microsporum canis* (n = 14)	TRB	0.03–0.125	0.09/0.125	0.07										7	5	2				
	ITZ	0.03–16	0.125/8.5	0.19			1				1	1	3	3	4	1				
	KTZ	0.125–16	0.5/20.5	0.93			2	1	1			3	3	4						
	FLZ	0.125–32	0.37/20	0.67		1		2		1	1	2	3	4						
	VCZ	0.125–16	0.5/16	0.86			2	1	1		1	3	5	1						
	PCZ	0.125–16	1.5/16	1.72			4	1	1	1	1	2	3	1						
	AMB	0.25–16	1/16	1.28			3		1	1	3	2	4							
*Nannizzia gypsea* (n = 6)	TRB	0.015–16	0.09/–	0.13			1							2	1	1	1			
	ITZ	0.125–8	0.37/–	0.70				2				1	1	2						
	KTZ	0.125–16	0.37/–	0.89			1	1				1	2	1						
	FLZ	0.125–16	0.62/–	0.62			1				1	1	2	1						
	VCZ	0.125–16	0.18/–	0.35			1						2	3						
	PCZ	0.25–16	1.25/–	1.58			3	1					2							
	AMB	0.125–8	0.37/–	0.70				1	1			1	2	1						
*Nannizzia fulva* (n = 2)	TRB	0.125–0.25	–/–	–									1	1						
	ITZ	0.25–0.5	–/–	–								1	1							
	KTZ	0.125–2	–/–	–						1				1						
	FLZ	0.25–1	–/–	–							1		1							
	VCZ	0.25–0.5	–/–	–								1	1							
	PCZ	0.5–1	–/–	–					1			1								
	AMB	0.5–1	–/–	–							1	1								

MIC, minimum inhibitory concentration; TRB, terbinafine; ITZ, itraconazole; KTZ, ketoconazole; FLZ, fluconazole; VCZ, voriconazole; PCZ, posaconazole; AMB, amphotericin B.

A comparison of MIC_50_ in II and II* genotypes of *T. interdigitale* showed that genotype II had a higher MIC_50_ with all drugs. In addition, isolates of XXIX genotype of *T. mentagrophytes* had a higher MIC_50_ as compared to *T. indotineae*.

In general, a comparison of *Trichophyton*, *Epidermophyton*, *Microsporum*, and *Nannizzia* genera showed that TRB and ITZ had the highest effect as compared to other drugs. *Trichophyton* genus had the lowest G mean against all drugs followed by *Nannizzia*. On the other hand, for all the drugs under study, *Epidermophyton* had the highest MIC_50_.

In *Nannizzia* genus, TRB and VCZ had a strong effect, KTZ had a weak effect, and AMB was inefficient (**Table 3**).

Among anthropophilic, geophilic, and zoophilic species, anthropophilic species had a high sensitivity to all drugs. In addition, the lowest and highest G mean in the anthropophilic group was observed in *T. rubrum* and *E. floccosum* isolates, respectively. On the other hand, AMB and PCZ had a low inhibitory effect against *E. floccosum* in the anthropophilic group. In general, geophilic and zoophilic species compared to anthropophilic species had a lower sensitivity to TRB and ITZ, respectively. Moreover, among zoophilic and geophilic species, *M. canis* and *N. gypsea* had a high level of sensitivity to TRB, respectively. Overall, azoles had the highest inhibitory effect against *T. tonsurans* species and the lowest inhibitory effect against *T. indotineae*.

Among all isolates, *T. indotineae* (n = 2), *T. mentagrophytes* (n = 1), *E. floccosum* (n = 1), *Trichophyton verrucosum* (n = 1), and *N. gypsea* (n = 1) demonstrated a decrease in sensitivity to TRB (MIC > 32 µg/ml). In addition, among all strains, two isolates (1.62%) were resistant to all drugs under study. The highest cross-resistance was observed between FLZ and ITZ (16.26%), and cross-resistance among azole antifungals was observed in 11 isolates (8.94%).

### 3.4 Point Mutation in *SQLE* Gene (Terbinafine-Resistant Strains)

**Figure 2:**
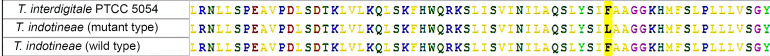
Comparison of the amino acid sequence of squalene epoxidase in terbinafine-sensitive strain and terbinafine-resistant strain.

To examine mutation in *SQLE* gene, all the six strains with a lower sensitivity to TRB were reproduced using Drsq primers and sequenced. The band length of the PCR product for *Trichophyton* was 500 bp, while it was 520 bp for *Epidermophyton* and *Nannizzia*. Forward and reverse sequences were edited using MEGA10 for each isolate, and then, the sequences were compared with sensitive strain sequences in GenBank using BLASTn. Afterward, the nucleic acid sequence was converted into an amino acid sequence. Among the six strains with a lower sensitivity to TRB, a point mutation was seen only in one strain of *T. indotineae* with MIC > 32, so Phe397Leu replacement of *SQLE* protein was observed. Replacement of C with A in *SQLE* gene leads to the replacement of Phe with Leu (**Figure 2**). The DNA sequence of the TRB-resistant isolate was recorded with accession no. OM373652 of *T. indotineae* in GenBank.

## 4 Discussion

In the light of continuing emergence of resistant dermatophytes to antifungal drugs around the world, monitoring drug resistance is essential (Siopi et al., 2021). In general, the identification of dermatophytes at the species level is a major issue for treating patients (Falahati et al., 2018). In this study, 123 clinical isolates of dermatophyte and five standard strains were examined. All the strains were identified by determining the ITS region sequence, and then the sensitivity pattern for seven antifungal drugs was determined using antifungal susceptibility testing (AFST). As the results showed, the number of men was twice the number of women, and tinea cruris was the dominant form. Other studies have reported tinea pedis and tinea capitis as the prevalent forms of dermatophytosis (Zareshahrabadi et al., 2020).

The current new taxonomy of dermatophytes separates *T. mentagrophytes* from its clonal offshoot *T. interdigitale* (Lipner and Scher, 2019). In fact, this classification is highly important from a clinical viewpoint, as *T. interdigitale* is only anthropophilic, and it is usually isolated from non-inflammatory cases such as tinea unguium and tinea pedis. In contrast, *T. indotineae* is mostly zoophilic and a cause of inflammatory symptoms, which mostly cause tinea corporis and tinea cruris. This is a key factor in selecting the right treatment (Taghipour et al., 2019; Siopi et al., 2021). In this study, *T. interdigitale* was the main causative agent of tinea pedis, while *E. floccosum* was responsible for most cases of tinea cruris. Siopi et al. reported that *T. interdigitale* species was mostly isolated from tinea pedis, which is similar to our results (Siopi et al., 2021).

In this study, the highest frequency of ITS genotypes of *T. interdigitale* and *T. mentagrophytes* belonged to II and VIII genotypes, respectively. Taghipour et al. showed that the VIII genotype was the most common in *T. mentagrophytes* species (Taghipour et al., 2019). In addition, a new XXIX genotype was found in *T. mentagrophytes* in this study. Dabas et al. showed that out of 123 dermatophyte isolates, 56% were *T. interdigitale*, which had been first mistakenly identified as *T. mentagrophytes*. Through determining the sequence of ITS regions, these two species were differentiated (Dabas et al., 2017), which is in accordance with our findings.

The phylogenetic tree using RAxML analysis showed that the sequence of ITS region can effectively differentiate dermatophyte species. Despite that Baert et al. failed to differentiate *Nannizzia* and *Epidermophyton* genera using the sequence of the ITS and BT2 regions, the results showed that the sequence of ITS regions can differentiate these two genera (Baert et al., 2020). Our results are consistent with Gräser et al. (1999).

According to the therapeutic protocols, dermatophytosis TRB is the first choice as a systemic treatment. Also, the latest studies showed the number of resistant cases to TRB is increasing (Dogra et al., 2017). *SQLE* gene mutation changes the protein structure and interrupts the drug attachment to the target enzyme (Shankarnarayan et al., 2020; Shaw et al., 2020; Kong et al., 2021). Here, six strains showed a low sensitivity to TRB, and among them, only one isolate (*T. mentagrophytes*) had a mutation on the Phe397Leu amino acid position. The cause of resistance in other strains might be replacement in other areas of *SQLE* gene or other intervening mechanisms of resistance.

According to Hiruma et al., the highest TRB resistance was observed in *T. rubrum* with L393F replacement in *SQLE* gene (Hiruma et al., 2021). Salehi et al. examined mutation in *SQLE* gene and reported replacement in Phe397Leu amino acid in *T. tonsurans* and *T. rubrum* species (Salehi et al., 2018b). In addition, Rezaei-Matehkolaei et al. showed *SQLE* gene mutation in five strains of TRB-resistant *T. interdigitale* and *T. mentagrophytes* (Rezaei-Matehkolaei et al., 2013). Singh et al. reported a replacement in Phe397Leu and Leu393Phe positions in TRB-resistant *T. interdigitale* strains (Singh et al., 2018). On the other hand, Yamada et al. found the replacement of amino acid in Phe397Leu position of *SQLE* gene in TRB-resistant *T. rubrum* species (Yamada et al., 2017). A similar examination was conducted by Lagowski et al. on resistant strains of *T. mentagrophytes*, and four strains with a mutation at the Leu393Phe region were reported (Lagowski et al., 2020).

In a study by Taghipour et al. of 45 strains of *T. mentagrophytes*, 5 strains (11.11%) were TRB resistant and had substitutions in Ala448Thr, Leu393Ser, and Phe397Leu positions in *SQLE* protein (Dabas et al., 2017). All the TRB-resistant isolates of *T. mentagrophytes* in other studies belonged to the VIII type of ITS genotype. In our study, out of 16 *T. mentagrophytes*, 3 strains were less sensitive to TRB, of which only 1 strain had a mutation in *SQLE* gene. This finding can be explained by the low number of *T. mentagrophytes* isolates or the low number of the VIII type isolates (n = 10). Also, in this study, contrary to the report by Heidemann et al. (2010), no significant correlation was seen between ITS genotype and clinical data, which is in line with the results of Salehi et al. It seems that conclusions about these data require further studies and samples.

In addition to TRB, AFST was carried out on six antifungal drugs. In general, AFST results of all isolates showed that, despite the resistant strains, TRB still is the most efficient drug against dermatophyte species. In addition, the results indicated that *T. indotineae* and *E. floccosum* had the lowest sensitivity to TRB. This finding is consistent with Lagowski et al. (2020), Rezaei-Matehkolaei et al. (2013), Rudramurthy et al. (2018), and Taghipour et al. (2019). Furthermore, in studies by Pourpak et al. (Pourpak and Firooz, 2021) and Kano et al. (2020), low sensitivity against TRB was reported in *T. indotineae*. Moreover, Taghipour et al. (2019) and Salehi et al. (2018b) showed that *T. interdigitale* species were more sensitive to TRB than *T. mentagrophytes* and *T. indotineae*, which is also consistent with our study.

In addition to TRB, ITZ is also prescribed for systemic treatment of dermatophytosis (Siopi et al., 2021). Here, ITZ had the highest and lowest effects on *M. canis* (G mean = 0.19 μg/ml) and *T. indotineae* (G mean = 0.9 μg/ml), respectively. On the other hand, FLZ demonstrated high levels of MIC, which is also consistent with Curatolo et al. (2020) and Barros et al. (2006). Curatolo et al. (2020) showed that TRB and ITZ had a good suppressive effect, which is consistent with the findings of the present study.

Aneke et al. (2018) reported that TRB and VCZ had the highest resistance effect on dermatophyte species and *M. canis* species in particular. This is not consistent with our finding of VCZ. It has been shown that in *T. mentagrophytes/T. interdigitale* complex, genotype XXIX of *T. mentagrophytes* and genotype II of *T. interdigitale* had a higher MIC_50_ with all drugs under study. This finding indicates that specific genotypes of this complex species had a higher MIC than others. Further examination of this topic can lead to the identification of more important species.

Among all tested isolates in our study, three strains were resistant to all drugs. In five strains (4.06%) out of all species, cross-resistance between TRB and other azoles drugs was observed, which is expected given that both groups of drugs suppress *SQLE* and cytochrome on the ergosterol biosynthesis path. It is suggested that in addition to major point mutations in *SQLE* gene (L393S, L393F, F397L, and Q408L), which were examined in the present study, other less frequent mutations including F415S, H440Y, and A448T are further investigated. Likewise, selecting more isolates from less frequent dermatophyte species of different geographic regions will help to provide more accurate data about antifungal susceptibility and genotype variations within the population.

## 5 Conclusion

With respect to the increasing prevalence of dermatophytosis and the growing numbers of antifungal drug resistance in dermatophyte species, precise identification of the etiologic species by molecular methods is believed to be crucial to achieve more effective treatments. In addition, epidemiological changes and lack of drug susceptibility testing have led to the failure of treatment and subsequent recurrence of the infection. Determining genotypes can improve our epidemiological information because specific genotypes have higher and different AFST and MIC results, which can help us to have a better diagnosis and treatment. Apparently, determining choice-effective drugs and drug-resistant strains through AFST can bring us closer to more efficient therapeutic goals. Given that TRB is the frontline defense against dermatophytosis, the growing resistance to TRB is a considerable challenge. Altogether, our results showed that precise identification of etiologic dermatophyte species and prescribing antifungal drugs with more caution can prevent resistance in strains, effectively reducing frequently recurrent infections, and prevent the distribution of the infection within the population.

## Data Availability Statement

The original contributions presented in the study are included in the article/**Supplementary Material**. Further inquiries can be directed to the corresponding author.

## Ethics Statement

The studies involving human participants were reviewed and approved by IR.PII.REC.1397.021. Written informed consent to participate in this study was provided by the participants’ legal guardian/next of kin.

## Author Contributions

NP performed the experiments, contributed to the analysis of the data, and drafted the manuscript. MS-G, ACN, MA and ZS assisted in the interpretation of the molecular data, data analysis and editing the manuscript. MR-A supervised the study, participated in the study design and data analysis, and approved the final draft. All authors have read and approved the final version of the manuscript.

## Funding

This work was supported financially by the Pasteur Institute of Iran.

## Conflict of Interest

The authors declare that the research was conducted in the absence of any commercial or financial relationships that could be construed as a potential conflict of interest.

## Publisher’s Note

All claims expressed in this article are solely those of the authors and do not necessarily represent those of their affiliated organizations, or those of the publisher, the editors and the reviewers. Any product that may be evaluated in this article, or claim that may be made by its manufacturer, is not guaranteed or endorsed by the publisher.
