# Supplementary material for: Phylogeny, Antifungal Susceptibility, and Point Mutations of SQLE Gene in Major Pathogenic Dermatophytes Isolated From Clinical Dermatophytosis
Source: Front Cell Infect Microbiol. 2022 Mar 18;12:851769. doi: 10.3389/fcimb.2022.851769 (PMC8972121; doi:10.3389/fcimb.2022.851769)
Supplement: Supplementary file 1 [file DataSheet_1.docx]

**Supplementary material**


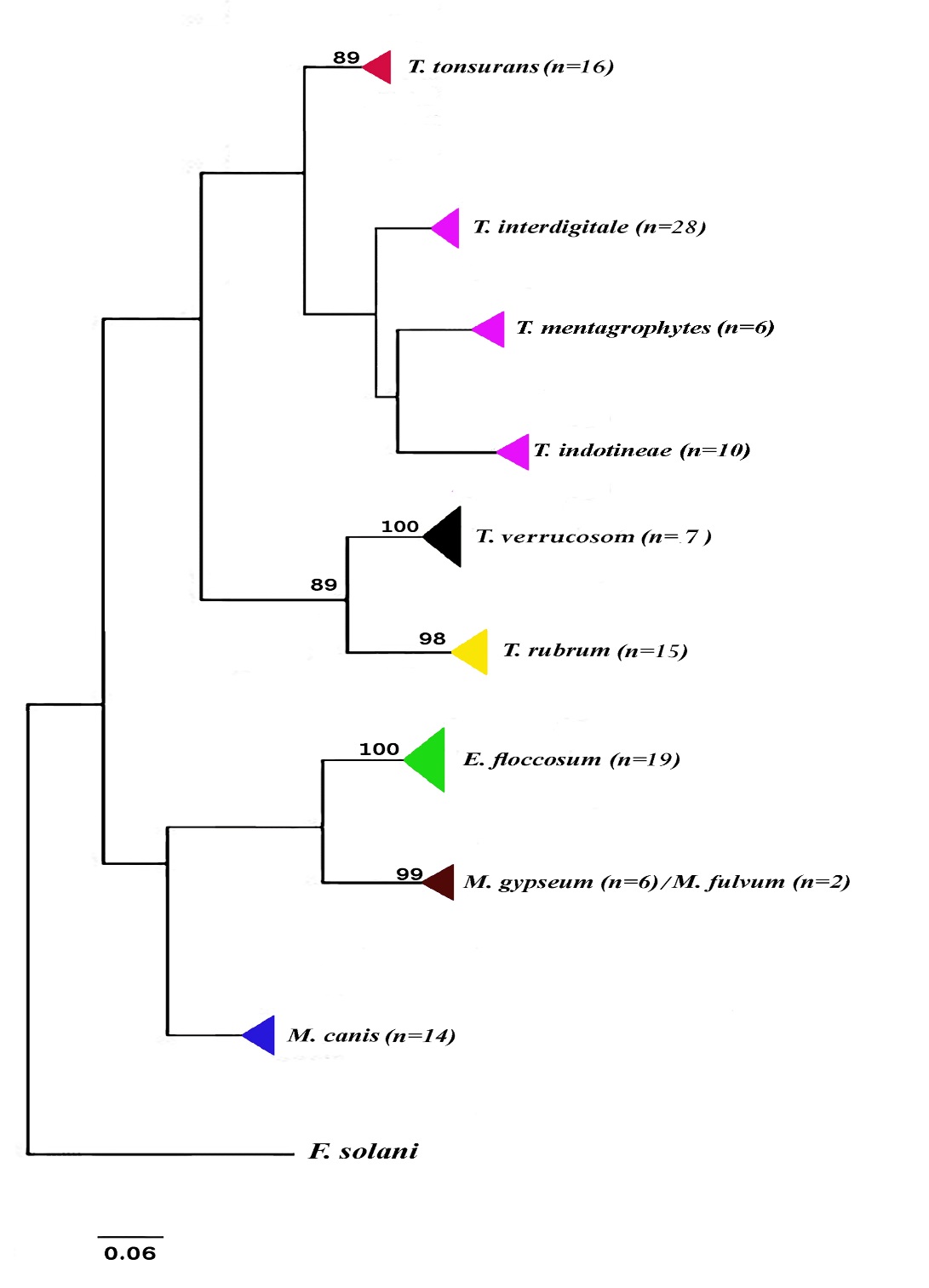


**Figure S1** Phylogenetic tree constructed by the RaxML analysis of the ITS of 123 dermatophyte isolates. *Fusarium* *solani* uses as an out-group. The bootstraps of more than 85% were indicated.
